# Educational associations with missed GP appointments for patients under 35 years old: administrative data linkage study

**DOI:** 10.1186/s12916-021-02100-7

**Published:** 2021-09-27

**Authors:** Ross McQueenie, David A. Ellis, Michael Fleming, Philip Wilson, Andrea E. Williamson

**Affiliations:** 1 Public Health Scotland, NHS Scotland, Meridian Court, 5 Cadogan Street, Glasgow, G2 6QE UK; 2 School of Management, University of Bath, Claverton Down, Bath, BA2 7AY UK; 3 Public Health, Institute of Health and Wellbeing, MVLS, University of Glasgow, 1 Lilybank Gardens, Glasgow, G12 8RZ UK; 4 Centre for Rural Health, Institute of Applied Health Sciences, University of Aberdeen, Old Perth Road, Inverness, IV2 3JH UK; 5 General Practice and Primary Care, School of Medicine, Dentistry and Nursing, MVLS, University of Glasgow, 1 Horselethill Road, Glasgow, G12 9LX UK

**Keywords:** Missed appointments, Engagement in health care, Education attendance, Education attainment, School exclusions, General practice, Primary care, Public health, Life course

## Abstract

**Background:**

There is an evidence gap about whether levels of engagement with public services such as schools and health care affect people across the lifespan. Data on missed patient appointments from a nationally representative sample of Scottish general practices (GP) (2013–2016) were probabilistically linked to secondary school pupil data. We tested whether school attendance, exclusions (2007–2011) or lower educational attainment (2007–2016) was associated with an increased risk of missing general practice appointments.

**Methods:**

School attendance data were classified into quartiles of possible days attended for years we had data. School exclusions were derived as a categorical variable of ‘ever excluded’. Attainment data were categorised via the Scottish Credit and Qualifications Framework (SCQF) level 3 or 6; a cumulative measure of attainment on leaving school. The associations between school attendance, exclusions and attainment and risk of missing medical appointments were investigated using negative binomial models, offset by number of GP appointments made and controlling for potential confounders.

**Results:**

112,534 patients (all aged under 35) had GP appointment and retrospective school attendance and exclusion data, and a subset of 66,967 also had attainment data available. Patients who had lower attendance, had been excluded from school or had lower educational attainment had an increased risk of missing GP appointments (all rate ratios > 1.40).

**Conclusions:**

This study provides the first evidence from a population-representative sample in a high-income country that increased numbers of missed appointments in health care are associated with reduced school attendance, higher levels of school exclusion and lower educational attainment. Insights into the epidemiology of missingness across public services can support future research, policy and practice that aim to improve healthcare, health outcomes and engagement in services.

## Background

People can be missing from care at multiple times and in multiple settings across their life. However, little is known about how levels of engagement with public services such as schools and health care are intertwined. We know that poverty and socio-economic inequality are strong predictors of life chances and health outcomes [1–3]. School attendance, exclusions and educational attainment are equally likely to be important factors [4]. Previous health service research has focussed on the association of single [5] or multiple health conditions with school attendance [6], or the association of school attainment with broader determinants of health outcomes such as life expectancy [7]. No previous studies have examined the role of engagement with education and health care specifically.

Health care systems themselves also play an important role in health outcomes [1–3]. These systems may encourage or discourage patients to engage in care: the characteristics of both services and patients interact to determine attendance rates [8]. Health care attendance is strongly associated with morbidity [9] and mortality. Patients who miss on average more than two GP appointments per year, for example, are much more likely than others to experience socio-economic deprivation and to have poorer health outcomes including a markedly increased risk of premature mortality [8, 10–12]. Urban general practices in affluent areas that typically have appointment waiting times of 2–3 days are the most likely to have patients who serially miss appointments [8].

Here, we test three hypotheses: (1) poor school attendance is predictive of poor health service attendance; (2) that being excluded from school is too; and [3] lower educational attainment is associated with high levels of missed general practice (GP) appointments. School exclusion is an extreme cause for non-attendance, usually triggered by disruptive behaviours. If these hypotheses are confirmed, our findings will further support existing evidence that high social complexity has a role in missingness in health care [8, 12], and this will have implications for future interventions development.

Data from Scotland allow these hypotheses to be tested because almost all of the population including children are registered with and receive health care from a GP practice. Scheduling appointments with a GP practice is under the control of patients (or their carers) whenever they seek care or are invited for a long-term conditions (LTC) review (such as for asthma annually). GPs act as the gatekeeper for all secondary care services (except emergency department visits, alcohol and drug recovery services and sexual health services). Ninety-five percent of the Scottish population receive their school education in the public sector, with assessment overseen by a single examinations board. The GP data were collected by a National Health Service (NHS) approved Trusted Third Party using established data collection and data processing routes as described previously [10]. The Scottish Government routinely collects population wide school data which can be made available to researchers on request [13].

## Methods

We used a large, retrospective sample (*n* = 824,374) of patient records from a nationally representative sample of Scottish general practices with codes extracted from clinically collected general practice data across Scotland over a 3-year period from September 2013 until September 2016. Requested data were extracted by the Trusted Third Party, anonymised and associated with a unique patient identifier in the National NHS Safehaven for analyses. Details of the data extraction are reported in previous publications [8, 10, 11].

Patients were descriptively categorised into general practice (GP) attendance categories based on their average number of missed appointments over the three-year study period: *zero missed appointments* (0 over the 3 year period), *low missed appointments* (< 1 per year on average), *medium missed appointments* (1–2 per year on average) and *high missed appointments* (> 2 per year on average) [8]. Categorisation in these groups was developed from qualitative and quantitative analysis of our pilot data [10], and subsequent papers describe a range of important demographic [8], health [11] and hospital utilisation outcomes [14]. However, counts of appointments (rather than categories) are used in later statistical models.

Permissions were obtained to link GP data (2013–2016) to the annually recorded Scottish Pupil Census which are held by the Scottish Exchange of Educational Data (ScotXeD) and which record anonymised pupil level data from all Scottish local authority funded primary, secondary and special schools. School attendance and exclusions data were obtained from 2007 to 2011 and attainment data from 2007 to 2016. Linkage was conducted by the National Records Scotland (NRS) indexing team using established methods to match pupil identifiers probabilistically to the Community Health Index (CHI) database [15]. Records can be matched using exact linkage, where a common unique identifier such as the CHI number is present on both records, or using probabilistic matching such as in this case, where such an identifier is not present or is of poor quality necessitating the use of alternative personal identifiers such as names, sex, date of birth and postcode. In probabilistic matching, records are bought together based on the ‘likelihood’ that they belong to the same person [16]. These data were then de-identified and imported securely to the National NHS Safehaven for the research team to analyse.

We analysed data for all patients in our original GP data set for whom education data were available. Availability was largely age dependent as data could only be linked from 2007 onwards and a minority of patients did not undertake their education in Scotland. School attendance was analysed in quartiles of percentage of days attended at school over the whole study period, calculated from the mean of the ratio of actual attendances to possible attendances for each year to take account of children only attending school in some of the years. Exclusions were classified as a categorical variable of ‘ever excluded’ (yes or no).

Attainment was recorded using the Scottish Credit and Qualifications Framework (SCQF) level 3 or 6. These are a cumulative measure of attainment derived on leaving school [17]. SCQF level 3 signifies a level of literacy and numeracy just below that required for Scottish Vocational Qualifications or apprenticeship eligibility. SCQF level 6 equates to a level of literacy and numeracy typically required to achieve university entry [18]; 61.6% of Scottish school leavers in 2016 had one SCQF level 6 qualification [19]. There is no direct measure of literacy or numeracy in the Scottish Pupil Census.

We performed negative binomial regression modelling using attendance, exclusions and attainment variables as predictors to quantify their respective associations with the outcome variable, number of missed GP appointments. Rate ratios quantified the risk of missing GP appointments. All negative binomial models were adjusted for age, sex, Scottish Index of Multiple Deprivation (SIMD) and number of long-term health conditions. SIMD is the standard measure of socio-economic deprivation at the small area level (data zones) used in policy and research in Scotland. It includes measures relating to income, employment, education, health, access to services, crime and housing [20].

Long-term conditions (LTC) were ascertained using patients’ primary care Read codes (used by GP practices) to code health problems and prescribing data [21]. LTC counts were generated for a previous paper [11] based on 43 long-term conditions as described by Barnett et al. [22] in their paper reporting patient level multi-morbidity in Scotland.

These models were offset for number of GP appointments scheduled in order to account for likely associations between numbers of scheduled and missed appointments.

## Results

**Fig. 1:**
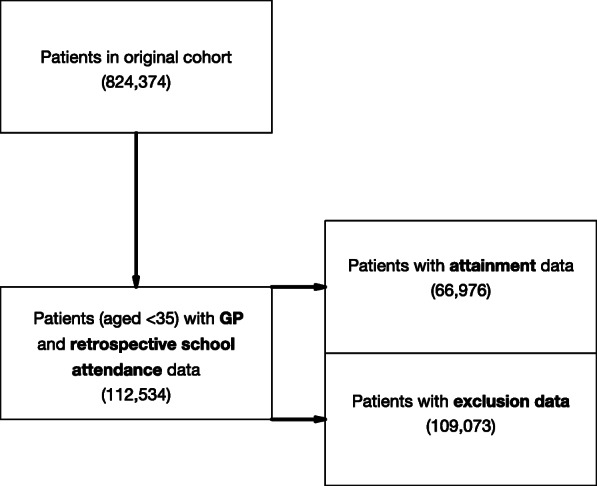
A flow diagram describes how the sample of participants was achieved

112,534/824,374 (14%) patients in the GP data set had school attendance and exclusions data and a smaller subset, 66,967 (8%) also had school-leaving attainment data available. Figure 1 is a flow diagram of participants included in the study.

The demographic characteristics of this sample in relation to GP missed appointment classification are described in Table 1.

**Table 1:** Age, sex, SIMD, long-term conditions, school exclusions, school attendance quartiles and SCQF 3 or 6 attainment by GP missed appointments category

Demographic factor	Missed appointment category	Number (percentage of category)			
	Zero	Low	Medium	High	Total
**Age**					
6–10	1884, [71.7%]	563, [21.4%]	141, [5.4%]	41, [1.6%]	2629, [100%]
11–14	17,118, [69.6%]	5735, [23.3%]	1347, [5.5%]	390, [1.6%]	24,590, [100%]
15–24	39,402, [52.8%]	22,069, [29.6%]	9219, [12.4%]	3918, [5.3%]	74,608, [100%]
25–34	4211, [58.1%]	2009, [27.7%]	731, [10.1%]	295, [4.1%]	7246, [100%]
**Sex**					
Female	30,696, [53.4%]	16,411, [28.5%]	7115, [12.4%]	3285, [5.7%]	57,507, [100%]
Male	31,919, [61.9%]	13,965, [27.1%]	4323, [8.4%]	1359, [2.6%]	51,566, [100%]
**SIMD**					
1	5669, [45%]	4016, [31.9%]	1985, [15.8%]	928, [7.4%]	12,598, [100%]
2	5095, [48%]	3388, [31.9%]	1451, [13.7%]	685, [6.5%]	10,619, [100%]
3	5582, [52.2%]	3203, [29.9%]	1345, [12.6%]	572, [5.3%]	10,702, [100%]
4	5263, [53.2%]	2890, [29.2%]	1203, [12.2%]	529, [5.4%]	9885, [100%]
5	5524, [55.4%]	2883, [28.9%]	1119, [11.2%]	452, [4.5%]	9978, [100%]
6	6378, [61.2%]	2753, [26.4%]	981, [9.4%]	314, [3%]	10,426, [100%]
7	7213, [62.4%]	3035, [26.2%]	950, [8.2%]	368, [3.2%]	11,566, [100%]
8	6024, [61.7%]	2598, [26.6%]	829, [8.5%]	310, [3.2%]	9761, [100%]
9	6338, [64.6%]	2465, [25.1%]	759, [7.7%]	250, [2.5%]	9812, [100%]
10	8137, [71.3%]	2551, [22.4%]	575, [5%]	143, [1.3%]	11,406, [100%]
**Number of long-term conditions (LTCs)**					
None	44,448, [65.8%]	17,139, [25.4%]	4751, [7%]	1208, [1.8%]	67,546, [100%]
One to three	18,060, [44.2%]	13,092, [32%]	6525, [16%]	3212, [7.9%]	40,889, [100%]
Four plus	107, [16.8%]	145, [22.7%]	162, [25.4%]	224, [35.1%]	638, [100%]
**Ever excluded from school**					
No	59,934, [59.1%]	27,747, [27.4%]	9915, [9.8%]	3782, [3.7%]	101,378, [100%]
Yes	2681, [34.8%]	2629, [34.2%]	1523, [19.8%]	862, [11.2%]	7695, [100%]
**School attendance quartile**					
1 (lowest attendance)	17,650, [63.4%]	7162, [25.7%]	2192, [7.9%]	814, [2.9%]	27,818, [100%]
2	16,271, [57.9%]	7889, [28.1%]	2794, [9.9%]	1131, [4%]	28,085, [100%]
3	17,267, [61.2%]	7571, [26.8%]	2579, [9.1%]	812, [2.9%]	28,229, [100%]
4 (highest attendance)	12,656, [44.6%]	8899, [31.3%]	4561, [16.1%]	2286, [8%]	28,402, [100%]
**SCQF level**					
3	7783, [38%]	6692, [32.7%]	3942, [19.2%]	2072, [10.1%]	20,489, [100%]
6	27,239, [58.6%]	13,292, [28.6%]	4478, [9.6%]	1472, [3.2%]	46,481, [100%]

After processing [8], the final dataset included information from 109,073 patients in total. A 3-year appointment history for each patient was uploaded to the NHS national secure safe haven (1,137,610 appointments). The mean age of included patients was 18.2 years (SD 4.44), 51,566 (47%) were male and 57,507 (53%) were female (Table 1). Seventy-five percent of the patients who had linked school attendance records were aged 15–34 years old, and measures of SIMD were evenly distributed across the school attendance sample. Thirty-eight percent of patients had 1–3 long-term health conditions, with 0.6% having four or more.

We note that only 6% of the sample who had attainment data were aged 11–15 years, likely due to small numbers leaving school before age 16. Of the attainment data sample, 55% were female and socio-economic deprivation measures were evenly distributed. We observed that 41% of these patients had 1–3 long-term health conditions and 0.8% had four or more.

### School attendance, exclusions and missed appointments

After offsetting for number of GP appointments scheduled and adjusting for age, sex, SIMD and number of long-term health conditions, a single percentage of increased attendance (over the entire study period) reduces the chance of missing a GP appointment by 1% (95% CI 0.98–0.99).

**Fig. 2:**
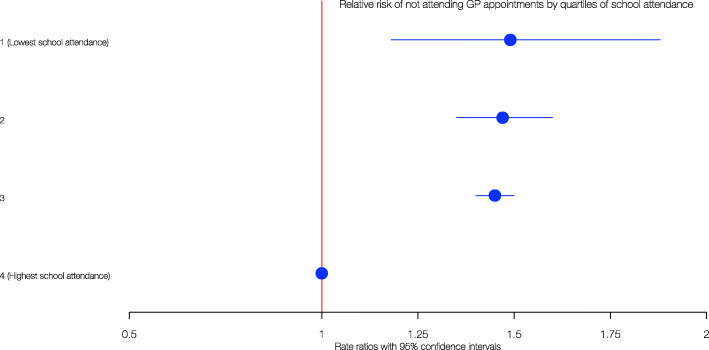
A Forest plot illustrating the relative risk of not attending GP appointments by quartiles of school attendance

Given the skewed distribution, quartiles of attendance were used to illustrate the differences over a longer period of time in an educational setting. When analysed in this way, patients with the lowest level of school attendance were 49% more likely (RR 1.49, 95% CI 1.18–1.88) to miss GP appointments compared with those in highest attendance quartile after adjusting for age, sex, SIMD, number of long-term conditions and number of GP appointments scheduled. This remained stable at RR 1.45 and 95% CI 1.40–1.50 and RR 1.47 and 95% CI 1.35–1.60 when comparing the highest attendance quartile with the third and second lowest quartiles respectively (Fig. 2).

Patients who had ever been excluded from school were also at increased risk of missing general practice appointments (RR 1.47, 95% CI 1.43–1.52) after adjusting for age, sex, SIMD and number of long-term conditions.

### Educational attainment and missed appointments

Compared to achieving SCQF 6, patients achieving SCQF3 when leaving school had 44% (RR 1.44, 95% CI 1.41–1.47) increased risk of missing GP appointments after adjusting for age, sex, SIMD, number of long-term conditions and number of GP appointments scheduled.

## Discussion

We have reported findings from a unique population-based dataset, linking general practice utilisation data with educational engagement and attainment. Poorer attendance at school was associated with high levels of missed GP appointments while being excluded from school was an equally strong predictor. Lower educational attainment on leaving school was also associated with a similarly increased risk of missing GP appointments.

This study provides the first evidence from a population-representative sample in a high-income country that patterns of missingness in health care are also found in the school setting. School absenteeism, especially where attendance drops below 75%, may be predictive of future absenteeism in other settings. However, while missingness in health care is associated with lower educational attainment, the overlapping time intervals of the data mean any notion of causality should be treated with caution. The key interpretation of these results is that factors that influence or shape engagement in care for an important minority of patients are complex. Future interventions to reduce missingness in health care should be developed in a way that takes such complexity into account.

In this study of 824,374 from patients across Scotland, 14% had school attendance data and 8% had attainment metrics available because data about attendance and attainment were only available for patients up to the age of 34. Therefore, our findings are restricted to younger patients, generally with low levels of morbidity. However, and more importantly, the data available was of a sufficient quality to be linked using probability matching (with an accuracy of 99%) whereby 95% of records were successfully matched [23].

The role that the family may have in influencing health service and school engagement patterns and the complexity of what may underpin our findings cannot be inferred from the available data. Access to data that would allow us to link siblings and children to their parents was declined by the data permissions panel because our study methodology was considered exploratory. Further research could explore this from the perspective of health service, education and other services engagement that impact on the social determinants of health for individuals.

Reviewing existing interventions to increase engagement in care across a range of sectors and translating these into novel settings is also worthy of further investigation. For example, electronic records, data sharing and risk identification may all have a role to play in identifying and responding to people who do not attend that are at high risk of adverse outcomes. Such approaches could be adopted both to develop interventions that reduce missingness in single sectors, such as primary care or school education, or across multiple social services.

## Conclusions

This study provides the first evidence from a population-representative sample in a high-income country that increased numbers of missed appointments in health care are associated with reduced school attendance, higher levels of school exclusion and lower educational attainment.

Insights into the epidemiology of missingness across public services can help researchers, planners and those working within the health service when designing future research, policy and practice that aims to improve engagement with services. At the same time, recognising the complexity of factors underlying low engagement and the role that engagement has across multiple aspects of peoples’ lives may be equally valuable when tackling health and associated social inequalities.

## Data Availability

These data were available from NHS Scotland and ScotXed. Permission for access was granted to the study team only from the participating GP practices, the Public Benefit and Privacy Panel NHS Scotland and Education Analytic Services, Scottish Government. Requests to access these data in the same manner as the authors can be made to http://www.escro.co.uk/ for general practice data; to https://www.isdscotland.org/Products-and-Services/eDRIS/ to host the analysis of general practice data and, for permissions and access to secondary care data; and to https://www.gov.scot/collections/scottish-exchange-of-data-scotxed/ for linked school education data. The authors did not have any special access privileges that other researchers would not have. Analysis code is available from the authors upon reasonable request. The code will replicate the study findings, but the analysis could also be replicated using other statistical software. Analysis code is available from the authors upon reasonable request. The code will replicate the study findings, but the analysis could also be replicated using other statistical software.
